# Analysis of the efficacy and safety of camrelizumab combined with chemotherapy for cervical cancer treatment

**DOI:** 10.3389/fonc.2025.1502701

**Published:** 2025-02-21

**Authors:** Song-Tian Yang, Shuang Yang, Ji Wang, Xiao-Ling Mu, Bo Yan

**Affiliations:** ^1^ College of Public Health, Chongqing Medical University, Chongqing, China; ^2^ First Affiliated Hospital of Chongqing Medical University, Chongqing, China; ^3^ College of Pharmacy, Chongqing Medical University, Chongqing, China

**Keywords:** cervical cancer, immunotherapy, camrelizumab, PD-1, combined therapy

## Abstract

**Introduction:**

In this study, we explored the effectiveness and safety of the immune checkpoint inhibitor camrelizumab in combination with chemotherapy for the treatment of cervical cancer by investigating adverse effects and changes in tumor volume, tumor activity, and biochemical indices.

**Methods:**

In this retrospective analysis, the experimental group was administered intravenous camrelizumab in conjunction with a TP regimen (consisting of a paclitaxel analog and a platinum analog), while the control group was given only the TP regimen. The effectiveness of solid tumors, SCC-Ag values, tumor volume, and apparent diffusion coefficient (ADC) values, as well as adverse responses, were compared between the two groups.

**Results:**

In the experimental group, 11 cases (22.45%) exhibited complete remission (CR), 27 cases (55.10%) demonstrated partial resolution (PR), and 11 cases (22.45%) showed stable disease (SD). The objective remission rate (ORR) was 77.55%, and the disease control rate (DCR) was 100%. In the control group, 10 cases (25.64%) exhibited CR, 18 cases (46.15%) demonstrated PR, 9 cases (23.08%) exhibited SD, and 2 cases (5.13%) exhibited disease progression. The ORR was 71.79%, and the DCR was 94.87%. A significant difference was observed between the two groups regarding the post-treatment SCC-Ag value, pre-treatment minimum ADC (minADC), and the change in size of minADC before and after treatment (P < 0.05). A comparison of the pre- and post-treatment size changes in tumor volume in patients with stage IIA2 cancer revealed significant differences (p < 0.05). No adverse responses of grade 3 or higher were detected.

**Discussion:**

Our analysis showed that the combination of camrelizumab and chemotherapy effectively reduced tumor size and malignancy in cervical cancer treatment, demonstrating robust anti-tumor activity with a favorable safety profile.

## Graphical Abstract

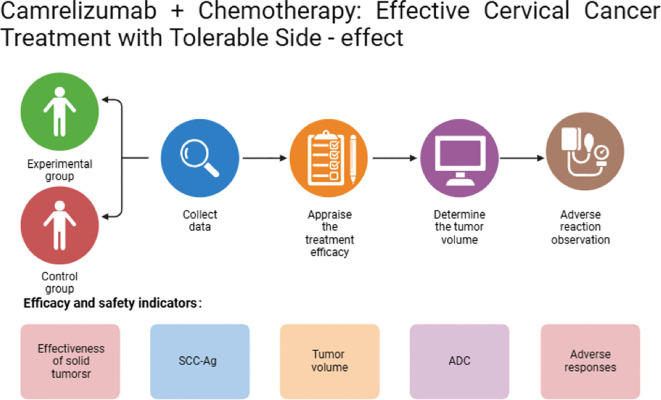

## 1 Introduction

Cervical cancer is one of the most prevalent malignant neoplasms among women, constituting a substantial health risk and acting as a leading cause of mortality in the female population. In 2022, 661,021 new cases of cervical cancer and 348,189 deaths were reported worldwide (1). In 2022, 150,700 new cases of cervical cancer and 55,700 deaths were reported in China (2).

In the treatment of cervical cancer, a suitable approach is determined on an individual basis, considering factors such as the patient’s clinical stage and tumor size. The principal integrated treatment modalities are surgery, radiotherapy, and chemotherapy (3). The prognosis is less favorable for patients presenting with advanced stage or large tumor size (4). For patients with larger tumors, chemotherapy is typically administered before surgery in an approach known as neoadjuvant chemotherapy (NACT), frequently leading to a positive therapeutic outcome and a favorable prognosis (5). However, a subset of patients with locally advanced cervical cancer may experience recurrence following chemotherapy treatment (6). Therefore, exploration of new treatment methods is necessary to improve the effectiveness of cervical cancer treatment.

The role of the immune system is an important aspect to consider in cervical cancer development and treatment. For example, human papillomavirus (HPV) infection is an etiological factor in the development of cervical cancer and is associated with a state of immune system compromise. In light of an immunocompromised state, individuals exhibit a pronounced susceptibility to HPV infection, which is often followed by the development of cervical cancer (7).The combination of immunotherapy with chemotherapy is a promising treatment option, and the use of immunotherapy is increasing. However, the therapeutic effectiveness of many immunological drugs is unknown.

Camrelizumab is an anti-PD-1 immune checkpoint inhibitor that received marketing approval in China in May 2019. In this study, we analyzed the efficacy and safety of camrelizumab in combination with chemotherapy by studying its biochemical indices, tumor volume, tumor malignancy, and adverse effects by studying clinicopathological data and magnetic resonance imaging (MRI) results.

## 2 Materials and methods

### 2.1 Patients

A retrospective analysis was performed on the clinical data of cervical cancer patients at the First Hospital of Chongqing Medical University from June 2022 to December 2023. The study was approved by the Ethics Committee of the First Affiliated Hospital of Chongqing Medical University, and informed consent was obtained from all patients (ethical approval number: 2020-425). The study was performed in accordance with the tenets of the Declaration of Helsinki.

Inclusion criteria: (1) Cervical cancer confirmed through histopathological examination, specifically including squamous carcinoma, adenocarcinoma, adenosquamous carcinoma, and a few other less common pathological forms. (2) Patients had not undergone radiation or chemotherapy prior to the relevant treatment course. (3) Prior to therapy, patients must have exhibited normal results in blood routine, coagulation function, and liver and renal function, as well as other relevant tests. In addition, they must not have had any hematological system disorders that impact fundamental vital signs. (4) A minimum of one observable abnormality is necessary. (5) Comprehensive gynecological examination and MRI results are required. (6) Patients were treated with either camrelizumab and TP regimen or with TP regimen.

Exclusion criteria: (1) lack of a single efficacy assessment; (2) incomplete clinical data; (3) presence of any other primary malignant tumor; (4) patients with immune diseases; (5) patients with a history of radiotherapy or chemotherapy; and (6) patients with previous hematology-related diseases.

### 2.2 Methods of treatment

The patients had received camrelizumab in conjunction with either a TP regimen or a TP regimen. Both groups were administered intravenous chemotherapy using the TP regimen and then had maintenance treatment every three weeks. The drug camrelizumab was intravenously administered in the experimental group at a dosage of 200 mg on days 1, 8, and 15 of a 3-week treatment cycle, followed by maintenance treatment every three weeks.

### 2.3 Evaluation of efficacy and adverse reactions

The effectiveness of the treatment was assessed using the standard method for evaluating the effectiveness of solid tumors, known as RECIST version 1.1. This standard defines efficacy as the achievement of complete remission (CR), partial resolution (PR), disease progression (PD), or stable disease (SD). The objective remission rate (ORR) is calculated by adding the proportion of CR and PR. A higher ORR signifies a larger proportion of patients who have achieved tumor reduction when undergoing the treatment. The disease control rate (DCR) is calculated by adding up the proportions of cases that have achieved CR, PR, and SD. The DCR quantifies the percentage of patients whose tumors were effectively managed and did not exhibit further progression. The presence of adverse reactions was assessed using the Common Terminology Criteria for Adverse Events (CTCAE) version 5.0.

### 2.4 Data collection

We retrieved various patient information from the hospital information system, including the name, age, pathological type, stage, virus type, first symptoms, medical history, MRI images, PD-L1 expression, SCC-Ag values, blood counts, liver and kidney function, thyroid function, and adverse events.

The MRI results were obtained using a GE HDxt 1.5T MRI scanner with an 8-channel body coil. The examination included T1-weighted imaging (T1WI), T2-weighted imaging (T2WI), and diffusion-weighted imaging (DWI). The imaging protocols included (1) axial: T1WI (GRE), T2WI (FRFSE), DWI (SE-EPI), and enhanced T1WI (LAVA); (2) coronal: T2WI (FRFSE) and enhanced T1WI (LAVA); (3) sagittal: T2WI (FRFSE) and enhanced T1WI (LAVA). The DWI parameters were as follows: repetition time/echo time = 7500/68 ms, layer thickness = 6 mm, field of view = 42 cm × 42 cm, matrix = 128 × 130, spacing = 2.5 mm, number of excitations = 4, and b = 0, 600, or 800 s/mm².

The apparent diffusion coefficient (ADC) values of the patient were assessed by two radiologists who had a decade of experience in analyzing gynecological MRI images. An agreement was achieved after each evaluation. In this work, each region of interest (ROI) were manually delineated on ADC images by identifying the maximum extent of cervical cancer lesions with an area ranging from 70 to 90 mm². The dimensions of each lesion were assessed on three separate occasions, and the average of these measurements was computed for the mean ADC (mADC), minimum ADC (minADC), and maximum ADC (maxADC) values of the three ROIs.

**Figure 1:**
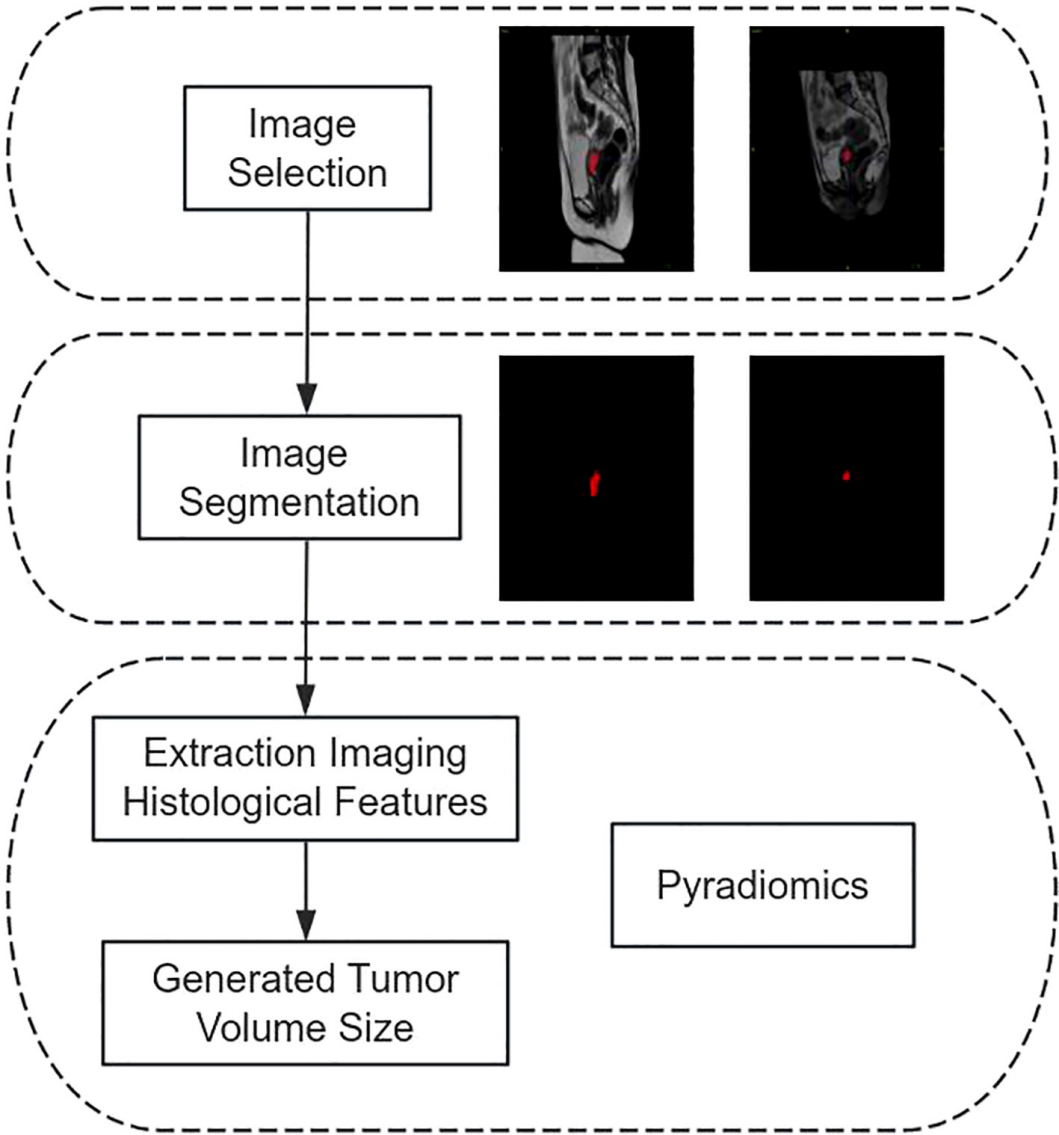
Flow chart of tumor volume measurement.

The patient’s tumor size was measured by importing pre- and post-treatment MRI pictures (T1, T2, and DWI sequences) from the hospital’s information system into the image processing program ITK-SNAP 3.8 (www.itksnap.org). Subsequently, a highly experienced gynecological oncologist (Doctor 1) meticulously analyzed the images, combining the ROI into a volume of interest. The photos were subsequently evaluated by a gynecological oncologist (Doctor 2), a highly experienced professional with more than twenty years of expertise in the field. Pyradiomics is utilized to extract histological information from medical images, which are then used to quantify the volumes of tumors before and after therapy. The flowchart illustrating the process of measuring tumor volume is shown in **Figure 1**.

### 2.5 Statistical methods

This study utilized the SPSS 26.0 program for statistical analysis. To evaluate the correlation between the patient’s factors and the effectiveness of the treatment, we utilized the χ2 test, Wilcoxon signed-rank test, and Mann–Whitney U test. The statistical significance criterion was established at p < 0.05.

## 3 Results

### 3.1 Population characteristic

Data were collected from a total of 156 cases of cervical cancer patients who received treatment at the First Affiliated Hospital of Chongqing Medical University from June 2022 to December 2023. After applying the criteria for inclusion and exclusion, a total of 88 cases were selected for the study. The experimental group consisted of 49 cases, while the control group consisted of 39 cases. The precise clinical attributes of the patients are outlined in **Table 1**.

**Table 1:** Patient Characteristics.

Clinical Characteristics	Experimental Group (n=49)	Control Group (n=39)	X^2^	P
Age				
≤50	14	6	2.235	0.327
50–60	20	20		
>60	15	13		
Pathology Type, n (%)				
Squamous cell carcinoma	41 (83.67%)	35 (89.75%)	15.957	0.001
Adenocarcinoma	6 (12.25%)	3 (7.69%)		
Neuroendocrine carcinoma	1 (2.04%)	1 (2.56%)		
Adenosquamous carcinoma	1 (2.04%)	0 (0.00%)		
Stage, n (%)				
IB2	1 (2.04%)	0 (0%)	9.304	0.677
IB3	1 (2.04%)	0 (0%)		
IIA1	3 (6.12%)	0 (0%)		
IIA2	11 (22.45%)	10 (25.64%)		
IIB	12 (24.50%)	9 (23.08%)		
IIIA	5 (10.21%)	4 (10.26%)		
IIIB	4 (8.16%)	2 (5.13%)		
IIIC1r	4 (8.16%)	6(15.38%)		
IIIC1p	2 (4.08%)	2 (5.13%)		
IIIC2r	2 (4.08%)	4 (10.26%)		
IIIC2p	1 (2.04%)	0 (0.00%)		
IVA	0 (0.00%)	1 (2.56%)		
IVB	3 (6.12%)	1 (2.56%)		
Surgery or not, n (%)				
Yes	36 (73.47%)	28 (71.79%)	0.031	0.861
No	13 (26.53%)	11 (28.21%)		
Tumors Expressed PD–L1, n (%)				
Negative	2 (4.08%)	0 (0%)	16.770	0.002
CPS<1	2 (4.08%)	0 (0%)		
1≤CPS<10	6 (12.24%)	0 (0%)		
CPS≥10	7 (14.29%)	0 (0%)		
Untested	32 (65.31%)	39 (100.0%)		

### 3.2 Evaluation of efficacy

The clinical effectiveness of the treatment was assessed using the RECIST (Response Evaluation Criteria in Solid Tumors) version 1.1 criteria, which are used to evaluate the effectiveness of anti-cancer treatments in patients with solid tumors. The experimental group exhibited 11 instances (22.45%) of CR, 27 instances (55.10%) of PR, and 11 instances (22.45%) of SD, resulting in an ORR of 77.55%. The DCR for this group was a perfect score of 100%. The control group exhibited 10 cases (25.64%) of CR, 18 cases (46.15%) of PR, 9 cases (23.08%) of SD, 2 cases (5.13%) of PD, and an ORR of 71.79%. The DCR for this group was 94.87%. Statistical analysis revealed a significant difference between the DCR of the experimental and control groups (P < 0.05). However, no statistically significant distinction was observed between the CR, PR, SD, PD, and ORR groups of the two cohorts (P > 0.05). To obtain comprehensive information about the essential details, please consult **Table 2**.

**Table 2:** Evaluation of Tumor Efficacy.

Tumor Efficacy	Experimental Group	Control Group	X^2^/Z	P
CR	11 (22.45%)	10 (25.64%)	0.275^b^	0.783
PR	27 (55.10%)	18 (46.15%)		
SD	11 (22.45%)	9 (23.08%)		
PD	0 (0.00%)	2 (5.13%)		
ORR	38 (77.55%)	28 (71.79%)	0.960^a^	0.327
DCR	49 (100.00%)	37 (94.87%)	5.128^a^	0.024

a.χ2 test b. Mann–Whitney U test.

The SCC-Ag values, tumor volume, mADC, minADC, and maxADC did not follow a normal distribution in both groups, as shown by the test. The Mann-Whitney U test and the Wilcoxon test were used to analyze the treatment effect indicators, which were presented as mean ± standard deviation. The specific information is displayed in **Table 3** below.

**Table 3:** Evaluation of Treatment Effects.

Type	Experimental Group	Control Group	Z	P
SCC–Ag (ng/ml)				
Pre–treatment	12.48 ± 19.13	10.80 ± 9.32	−0.605	0.545
Post–treatment	2.32 ± 2.30	2.96 ± 2.53	−2.119	0.028
Magnitude of the change	−10.20 ± 17.70	−7.34 ± 8.27	−0.067	0.946
Z	−5.826	−5.134		
P	0.000	0.000		
Tumor Volume (cm^3^)				
Pre–treatment	33.58 ± 31.33	30.81 ± 18.64	−0.760	0.447
Post–treatment	3.53 ± 5.41	3.67 ± 5.86	−0.572	0.567
Magnitude of the change	−30.05 ± 30.32	−27.15 ± 19.09	−0.517	0.605
Z	6.093	5.415		
P	0.000	0.000		
minADC (×10^–3^ mm^2^/s)				
Pre–treatment	314.09 ± 473.96	469.248 ± 434.25	−4.108	0.000
Post–treatment	616.07 ± 682.89	568.128 ± 716.92	−0.453	0.651
Magnitude of the change	302.20 ± 869.48	98.657 ± 724.11	−2.067	0.039
Z	–2.184	–0.363		
P	0.029	0.717		
maxADC (×10^–3^ mm^2^/s)				
Pre–treatment	622.58 ± 993.99	378.33 ± 656.87	−1.005	0.315
Post–treatment	1189.27 ± 1282.02	1018.64 ± 1153.27	−0.910	0.363
Magnitude of the change	566.65 ± 1712.10	640.29 ± 1032.65	−0.117	0.907
Z	−2.246	−3.140		
P	0.025	0.002		
mADC (×10^–3^ mm^2^/s)				
Pre–treatment	484.24 ± 759.25	277.22 ± 325.49	−0.848	0.397
Post–treatment	934.24 ± 1017.25	779.64 ± 906.42	−0.964	0.335
Magnitude of the change	450.00 ± 1345.26	502.42 ± 828.49	−0.067	0.946
Z	−2.274	−2.819		
P	0.023	0.005		

The National Comprehensive Cancer Network (NCCN) clinical guideline recommendations advise using radiographic imaging to assess stage IIA2 tumors (3). Stage IIA2 cervical cancer tumors are classified as locally advanced tumors. For individuals with this type of cervical cancer, concomitant radiation is typically deemed the most suitable therapeutic choice. To have a deeper understanding of the effectiveness of camrelizumab when used in conjunction with chemotherapy for treating cervical cancer, we have decided to reevaluate the tumor volume and ADC values for stage IIA2 cancer. The specific information is displayed in **Table 4** below.

**Table 4:** Evaluation of Tumor Volume and ADC Values in Stage IIA2 Cancer.

Type	Experimental Group	Control Group	Z	P
Tumor Volume (cm^3^)				
Pre–treatment	35.12 ± 33.87	17.41 ± 12.86	−1.690	0.099
Post–treatment	1.81 ± 2.67	5.68 ± 9.26	−0.671	0.512
Magnitude of the change	−33.31 ± 34.51	−11.73 ± 9.98	−2.253	0.024
Z	−2.934	−2.701		
P	0.003	0.007		
minADC (×10^–3^ mm^2^/s)				
Pre–treatment	128.27 ± 55.17	447.20 ± 319.13	−3.239	0.001
Post–treatment	594.55 ± 433.54	616.20 ± 831.91	−0.845	0.426
Magnitude of the change	466.61 ± 441.77	168.97 ± 837.85	−2.113	0.036
Z	−2.667	−0.357		
P	0.008	0.721		
maxADC (×10^–3^ mm^2^/s)				
Pre–treatment	211.55 ± 65.11	327.60 ± 205.59	−1.408	0.173
Post–treatment	1226.45 ± 1037.20	1118.30 ± 1350.14	−1.127	0.282
Magnitude of the change	1014.82 ± 1062.99	790.58 ± 1354.74	−1.127	0.282
Z	−2.845	−1.478		
P	0.004	0.139		
mADC (×10^–3^ mm^2^/s)				
Pre–treatment	178.45 ± 59.47	256.18 ± 152.96	−0.986	1.014
Post–treatment	959.06 ± 780.54	879.51 ± 1091.39	−0.986	0.349
Magnitude of the change	780.61 ± 798.34	623.33 ± 1090.10	−1.197	0.251
Z	−2.845	−1.172		
P	0.004	0.241		

### 3.3 Adverse reactions

According to the Common Terminology Criteria for Adverse Events (CTCAE) version 5.0, both the experimental and control groups encountered adverse reactions of different intensities. The statistical analysis showed a notable disparity in the occurrence of liver and renal function impairments between the two groups (P < 0.05). No statistically significant differences were observed in the occurrence of anemia, hypoproteinemia, hypokalemia, myelosuppression, and leukopenia (P > 0.05). The specific information is provided in **Table 5** below.

**Table 5:** Comparison of Adverse Reactions in the Two Patient Groups.

Adverse Reaction	Experimental Group		Control Group		X^2^	P
	Grade I–II	Grade III–V	Grade I–II	Grade III–V		
Anemia	5 (10.20%)	0	3 (7.69%)	0	0.244	0.621
Hypoproteinemia	2 (4.08%)	0	1 (2.56%)	0	0.148	1.000
Hypokalemia	1 (2.04%)	0	1 (2.56%)	0	0.205	1.000
Myelosuppression	3 (6.12%)	0	3 (7.69%)	0	0.307	0.579
Leucopenia	2 (4.08%)	0	3 (7.69%)	0	1.418	0.234
Abnormal liver function	9 (18.37%)	0	3 (7.69%)	0	4.421	0.036
Abnormal renal function	3 (6.12%)	0	7 (17.95%)	0	6.818	0.009
Abnormal thyroid function	7 (14.29%)	0	0	0	/	/
Thrombocytopenia	4 (8.16%)	0	0	0	/	/
Skin rash	3 (6.12%)	0	0	0	/	/
Phlebitis	1 (2.04%)	0	0	0	/	/

## 4 Discussion

In this study, we confirmed that the combination of camrelizumab and chemotherapy represents an effective approach for the treatment of cervical cancer, with the potential to reduce tumor volume and malignancy in patients with cervical cancer, without increasing the incidence of adverse effects. The ORR and DCR were 77.55% and 100%, respectively.

With the continuous improvement of therapeutic agents and regimens, NACT has become an important part of the cervical cancer. Despite ongoing debates, Despite ongoing debates, it has increasingly been the subject of research (8, 9). It facilitates the reduction of tumor size, lymph node metastasis, and paracervical infiltration (10–12). Consequently, it is still being used in clinical practice (12, 13).

The experimental group had a higher ORR (77.55% vs 71.79%) and DCR (100% vs 94.87%) than the control group. In cancer treatment, the combination of immunotherapy and chemotherapy has been demonstrated to be efficacious (14–18). The addition of camrelizumab is likely to enhance the anti - tumor effects (19, 20). This combinatorial therapeutic approach can more precisely target cancer cells and intensify the immune response of the body, thereby contributing to a significantly elevated response rate.

SCC-Ag represents the optimal tumor marker test for the diagnosis of cervical cancer and serves as a crucial indicator for the assessment of the outcomes of cervical cancer patients (21). After treatment, the SCC-Ag readings after treatment were considerably lower in the experimental group compared to those of the control group (2.32 ng/mL vs 2.96 ng/mL). Given that immunotherapy has the potential to more effectively suppress tumor growth, it is likely to result in a more substantial reduction in SCC - Ag, signifying a decreased risk of tumor recurrence (22).

A previous study demonstrated a link between tumor size and survival (23). The results of the present study demonstrated a significant decrease in tumor volume in both groups, with a substantial reduction observed. These results are comparable to the findings in resectable gastric/gastro - oesophageal junction cancer, where immunotherapy shrank tumors (24). Direct surgical treatment is challenging for patients with stage IIA2 cervical carcinoma owing to the size of the local tumor. Studies have shown that neoadjuvant chemotherapy before surgery effectively shrinks local tumor growth, making it easier for surgeons to remove the tumor. This approach also meets the needs of patients undergoing surgery (25). Our study revealed that patients with stage IIA2 tumors who were in the experimental group experienced a significant decrease in tumor volume when compared to that of the control group. The addition of camrelizumab may enhance this shrinking effect, possibly by promoting tumor cell apoptosis or inhibiting tumor angiogenesis, making it a more effective option for patients with large local tumors.

Pelvic MRI and Diffusion-weighted imaging have prognostic significance for the therapeutic effectiveness of cervical cancer and can be utilized to assess the early therapeutic response of cervical cancer (26).

The ADC values derived from DWI allow researchers to detect the presence of residual tumors and differentiate between the malignant grades of such tumors (27, 28). The ADC can be used to indicate changes in the density of tumor cells. When tumor cells undergo apoptosis, the density of tumor cells decreases and the ADC value increases (29). The mADC, minADC, and maxADC values of the experimental group showed a notable rise after post-treatment. Moreover, there was a noticeable and statistically significant difference in the extent of change between the two groups for the minADC pre-treatment and pre-treatment. This is also the case for the IIA2 stage results. As the ADC can indicate tumor cell density changes, the combination of camrelizumab and the TP regimen may be more effective in reducing tumor malignancy and promoting tumor cell specialization, perhaps by enhancing the immune - mediated killing of cancer cells.

Regarding safety, patients in both groups experienced adverse reactions classified as grade I–II, with no observed grade III–IV adverse reactions. All patients showed improvement in grade I–II adverse reactions following symptomatic post-treatment. Therefore, the treatment’s overall effectiveness was satisfactory.

Adverse reactions in the experimental group during the treatment period were found to be comparable to those documented in previous studies (30). The observed conditions comprised atypical thyroid activity, low platelet count, and skin irritation. Nevertheless, none of these reactions led to severe adverse events or mortality.

Upon conducting a statistical analysis of the adverse reactions, we found a significant difference between the two groups in terms of hepatic and renal function abnormalities (18.37% vs. 7.69%; 6.12% vs. 17.95%). In this study, we observed that the incidences of anemia, hypoproteinemia, and liver function abnormalities were higher in patients who underwent combination therapy than in those who received chemotherapy alone. This can be attributed to the cumulative toxicity of combination chemotherapy. In this study, the occurrence of anemia, hypoproteinemia, and liver function abnormalities was higher in patients who underwent combination therapy compared to those who received chemotherapy alone. The superimposed toxicity of combination chemotherapy may be the cause of this combination of adverse effects.

## 5 Conclusions

This study demonstrates that the combination of camrelizumab and chemotherapy is an effective treatment for cervical cancer, capable of reducing tumor size, attenuating malignancy, and showing robust anti-tumor activity with tolerable adverse effects. To facilitate the emergence of additional insights, future studies should increase the number of case studies and prolong the follow-up period. This will facilitate more comprehensive analysis and interpretation of the findings.

## Data Availability

The raw data supporting the conclusions of this article will be made available by the authors, without undue reservation.

## References

[1] Bray F Laversanne M Sung H Ferlay J Siegel RL Soerjomataram I. Global cancer statistics 2022: GLOBOCAN estimates of incidence and mortality worldwide for 36 cancers in 185 countries. CA: A Cancer J Clin. (2024) 74:229–63. doi: 10.3322/caac.21834 38572751

[2] Han B Zheng R Zeng H Wang S Sun K Chen R. Cancer incidence and mortality in China, 2022. J Natl Cancer Cent. (2024) 4:47–53. doi: 10.1016/j.jncc.2024.01.006 39036382 PMC11256708

[3] National Comprehensive Cancer Network. NCCN clinical practice guidelines in oncology (NCCN guidelines^®^) Cervical Cancer. Available online at: www.nccn.org/patients (Accessed June 15, 2018).

[4] Bhatla N Aoki D Sharma DN Sankaranarayanan R. Cancer of the cervix uteri: 2021 update. Int J Gynecol Obstet. (2021) 155:28–44. doi: 10.1002/ijgo.v155.s1 PMC9298213 34669203

[5] Ye Q Yuan H–X Chen H–L. Responsiveness of neoadjuvant chemotherapy before surgery predicts favorable prognosis for cervical cancer patients: a meta–analysis. J Cancer Res Clin Oncol. (2013) 139:1887–98. doi: 10.1007/s00432-013-1509-y PMC11824783 24022086

[6] Serour GI. A vision for FIGO 2009–2012. Int J Gynecol Obstet. (2010) 108:93–6. doi: 10.1016/j.ijgo.2009.11.001 19951817

[7] Hollington M. Cox proportional hazard–model application: time to cervical cancer screening among women living with HIV in South Africa. Infect Agent Cancer. (2024) 19:6. doi: 10.1186/s13027-023-00527-6 38431636 PMC10909268

[8] Herod J Burton A Buxton J Tobias J Luesley D Jordan S. A randomised, prospective, phase III clinical trial of primary bleomycin, ifosfamide and cisplatin (BIP) chemotherapy followed by radiotherapy versus radiotherapy alone in inoperable cancer of the cervix. Ann Oncol. (2000) 11:1175–82. doi: 10.1023/A:1008346901733 11061615

[9] de Azevedo CRAS Thuler LCS de Mello MJG de Oliveira Lima JT da Fonte ALF Fontão DFS. Phase II trial of neoadjuvant chemotherapy followed by chemoradiation in locally advanced cervical cancer. Gynecol Oncol. (2017) 146:560–5. doi: 10.1016/j.ygyno.2017.07.006 28709705

[10] Miriyala R Mahantshetty U Maheshwari A Gupta S. Neoadjuvant chemotherapy followed by surgery in cervical cancer: Past, present and future. Int J Gynecol Cancer. (2022) 32:260–5. doi: 10.1136/ijgc-2021-002531 35256411

[11] Mousavi A Gilani MM Akhavan S Hasani SS Alipour A Gholami H. The outcome of locally advanced cervical cancer in patients treated with neoadjuvant chemotherapy followed by radical hysterectomy and primary surgery. Iranian J Med Sci. (2021) 46:355. doi: 10.30476/ijms.2020.81973.0 PMC8438343 34539010

[12] Chen H Liang C Zhang L Huang S Wu X. Clinical efficacy of modified preoperative neoadjuvant chemotherapy in the treatment of locally advanced (stage IB2 to IIB) cervical cancer: A randomized study. Gynecol Oncol. (2008) 110:308–15. doi: 10.1016/j.ygyno.2008.05.026 18606439

[13] Benson R Pathy S Kumar L Mathur S Dadhwal V Mohanti BK. Locally advanced cervical cancer–neoadjuvant chemotherapy followed by concurrent chemoradiation and targeted therapy as maintenance: a phase II study. J Cancer Res Ther. (2019) 15:1359–64. doi: 10.4103/jcrt.JCRT_39_18 31898673

[14] Lan C Shen J Wang Y Li J Liu Z He M. Camrelizumab plus apatinib in patients with advanced cervical cancer (CLAP): a multicenter, open–label, single–arm, phase II trial. J Clin Oncol. (2020) 38:4095–106. doi: 10.1200/JCO.20.01920 PMC7768345 33052760

[15] PDQ Adult Treatment Editorial Board. Cervical cancer treatment (PDQ^®^): health professional version. In: PDQ Cancer Information Summaries. (2024) National Cancer Institute (US). Available at: https://pubmed.ncbi.nlm.nih.gov/26389493/.

[16] Li K Chen J Hu Y Wang Y–Z Shen Y Chen G. Neoadjuvant chemotherapy plus camrelizumab for locally advanced cervical cancer (NACI study): a multicentre, single–arm, phase 2 trial. Lancet Oncol. (2024) 25:76–85. doi: 10.1016/S1470-2045(23)00531-4 38048802

[17] Li G Cheng M Hong K Jiang Y. Clinical efficacy and safety of immunotherapy retreatment in metastatic cervical cancer: A retrospective study. OncoTargets Ther. (2023) 16:157–63. doi: 10.2147/OTT.S400376 PMC9999713 36911534

[18] Butterfield LH Najjar YG. Immunotherapy combination approaches: mechanisms, biomarkers and clinical observations. Nat Rev Immunol. (2024) 24:399–416. doi: 10.1038/s41577-023-00973-8 38057451 PMC11460566

[19] Shi W Liu N Lu H. Advancements and challenges in immunocytokines: A new arsenal against cancer. Acta Pharm Sin B. (2024) 14:4649–64. doi: 10.1016/j.apsb.2024.07.024 PMC11628837 39664443

[20] Song H Liu X Jiang L Li F Zhang R Wang P. Current status and prospects of camrelizumab, A humanized antibody against programmed cell death receptor 1. Recent Pat Anticancer Drug Discovery. (2021) 16:312–32. doi: 10.2174/22123970MTE09MDYg0 33563158

[21] Liu Z Shi H. Prognostic role of squamous cell carcinoma antigen in cervical cancer: A meta–analysis. Dis Markers. (2019) 2019:6710352. doi: 10.1155/2019/6710352 31275450 PMC6589214

[22] Fu J Wang W Wang Y Liu C Wang P. The role of squamous cell carcinoma antigen (SCC Ag) in outcome prediction after concurrent chemoradiotherapy and treatment decisions for patients with cervical cancer. Radiat Oncol. (2019) 14:1–5. doi: 10.1186/s13014-019-1355-4 31416463 PMC6694518

[23] Dercle L Sun S Seban R–D Mekki A Sun R Tselikas L. Emerging and evolving concepts in cancer immunotherapy imaging. Radiology. (2023) 306:32–46. doi: 10.1148/radiol.210518 36472538

[24] Janjigian YY Van Cutsem E Muro K Wainberg Z Al–Batran S–E Hyung WJ. MATTERHORN: phase III study of durvalumab plus FLOT chemotherapy in resectable gastric/gastroesophageal junction cancer. Future Oncol. (2022) 18:2465–73. doi: 10.2217/fon-2022-0093 35535555

[25] Mandic A Maricic S Malenkovic G Stojic I Gutic B. Neoadjuvant chemotherapy in locally advanced cervical cancer in pregnancy–Review of the literature. JBUON. (2020) 25:597–604. 32521840

[26] Gui B Micco M Valentini A Cambi F Pasciuto T Testa A. Prospective multimodal imaging assessment of locally advanced cervical cancer patients administered by chemoradiation followed by radical surgery—the “PRICE “study 2: role of conventional and DW–MRI. Eur Radiol. (2019) 29:2045–57. doi: 10.1007/s00330-018-5768-5 30324389

[27] Gu K–W Kim CK Choi CH Yoon YC Park W. Prognostic value of ADC quantification for clinical outcome in uterine cervical cancer treated with concurrent chemoradiotherapy. Eur Radiol. (2019) 29:6236–44. doi: 10.1007/s00330-019-06204-w 30980126

[28] Nougaret S Reinhold C Alsharif SS Addley H Arceneau J Molinari N. Endometrial cancer: combined MR volumetry and diffusion–weighted imaging for assessment of myometrial and lymphovascular invasion and tumor grade. Radiology. (2015) 276:797–808. doi: 10.1148/radiol.15141212 25928157 PMC5410943

[29] Li S Liu J Zhang F Yang M Zhang Z Liu J. Novel T2 mapping for evaluating cervical cancer features by providing quantitative T2 maps and synthetic morphologic images: a preliminary study. J Magnet Reson Imaging. (2020) 52:1859–69. doi: 10.1002/jmri.27297 32798294

[30] Xu J Shen J Gu S Zhang Y Wu L Wu J. Camrelizumab in combination with apatinib in patients with advanced hepatocellular carcinoma (RESCUE): a nonrandomized, open–label, phase II trial. Clin Cancer Res. (2021) 27:1003–11. doi: 10.1158/1078-0432.CCR-20-2571 33087333

